# Inhibition of Cortical Evoked Responses to Sound Pulses by Preceding Silent Gaps

**DOI:** 10.1007/s10162-025-00999-w

**Published:** 2025-07-23

**Authors:** Payam S. Shabestari, Niklas K. Edvall, Mikkel C. Vinding, Sven Vanneste, Daniel Lundqvist, Patrick Neff, Christopher R. Cederroth

**Affiliations:** 1 Department of Otorhinolaryngology, Head and Neck Surgery, University Hospital Zurich, University of Zurich, Zurich, Switzerland; 2 Laboratory of Experimental Audiology, Department of Physiology and Pharmacology, Karolinska Institute, Stockholm, Sweden; 3 NatMEG, Department of Clinical Neuroscience, Karolinska Institutet, Stockholm, Sweden; 4 Department of Psychology, University of Copenhagen, Copenhagen, Denmark; 5 Lab for Clinical and Integrative Neuroscience, Trinity Institute for Neuroscience, School of Psychology, Trinity College Dublin, Dublin, Ireland; 6 Department of Clinical Neuroscience, Karolinska Institutet, Stockholm, Sweden; 7 Department of Psychiatry and Psychotherapy, University of Regensburg, Regensburg, Germany; 8 Translational Hearing Research, Tübingen Hearing Research Center, Department of Otolaryngology, Head and Neck Surgery, University of Tübingen, Tübingen, Germany

**Keywords:** Magnetoencephalography, Auditory, Evoked response, Cortical inhibition, GPIAS

## Abstract

**Purpose:**

The basic principle of sensorimotor gating (SMG) relies on the ability of a weak lead stimulus (such as a pre-pulse) to inhibit a startling effect of a following, more intense, abrupt stimulus—the so-called pre-pulse inhibition (PPI) paradigm. PPI has been used for near half a century as a means to investigate psychiatric disorders in which its disruption is a surrogate for altered SMG in schizophrenia. However, the blinking response is very variable, making it a poor outcome measure at the individual level. Unlike PPI, which is regulated in the lateral globus pallidus from the basal ganglia, inhibition of the startle reflex by preceding silent gaps embedded in continuous background noise is processed in the auditory cortex, making it particularly suitable for measuring cortical responses.

**Methods:**

Here, based on the behavioral gap-pre-pulse inhibition of acoustic startle (GPIAS) stemming from animal research in tinnitus research, we present a new sensory gating (SG) paradigm using source-localized magnetoencephalography (MEG) in 26 normal hearing healthy participants (13 females, 12 males, 1 other) with a mean age of 28.4 (SD $\pm 5.8$), where we expose them to various levels of sound pulses in presence or absence of preceding silent gaps embedded in broadband carrier noises of either 60 or 70 dB SPL, using various interstimulus intervals (ISI: 0, 60, 120, 240 ms).

**Results:**

We evidence a near 72.5% (SD $\pm 15.9$) suppression of N1 evoked response to a pulse as high as 90 dB(A) sound pressure level (SPL) when preceded by a 50 ms long silent gap in a 60 dB(A) SPL broadband carrier noise. Cortical inhibition was greatest with 240 ms ISI between gap and pulses, and about 1.5 times larger in the right transverse temporal gyrus when compared to the left hemisphere. While merely 68% of the individuals blinked at the highest pulse levels, cortical evoked responses were found in all participants.

**Conclusion:**

Overall, we provide evidence that SG, measured by N1 cortical response to sound pulses, is reliably inhibited by preceding gaps. We propose this paradigm as an effective method to assess auditory SG through development and aging, and potentially as a method for the diagnosis of hearing disorders like tinnitus or hyperacusis.

**Supplementary Information:**

The online version contains supplementary material available at 10.1007/s10162-025-00999-w.

## Introduction

Sensorimotor gating (SMG) has been investigated for more than half a century to understand inhibitory mechanisms in the brain. The basic principle of SMG relies on the ability of a weak lead stimulus (a pre-pulse) to inhibit a startling effect of a following, more intense, abrupt stimulus—the so-called pre-pulse inhibition (PPI) paradigm. Silent gaps embedded in a broadband carrier noise also have such ability when preceding a brief startling pulse (the gap pre-pulse inhibition of the startle response or GPIAS). In the clinical context, these paradigms have been used as an automatic or pre-conscious inhibition of the motor reflex response that occurs in healthy subjects [29]. In humans, this SMG is measured by the amplitude of the electromyograms (EMG) inferring on the degree of contraction of the orbicularis oculi muscle whereas in rodents, this is measured by amplitude of the whole-body flinch causing pressure on a piezo sensor, or a force transducer.

While PPI has been mainly used to evidence SMG impairment in patients with schizophrenia [6, 7], gap detection (e.g., gap-in-noise, random gap detection test) has been used to understand the neural coding of transient amplitude shifts in the sound envelope as a measure of temporal resolution in the auditory domain [47, 48]. For instance, this temporal resolution is situated, in both animals and humans, around a duration of 2 ms in comfortably loud broadband noise (BBN) up to 20 ms at intensities near hearing threshold. In the last decade, GPIAS, which uses the ability of silent gaps to inhibit a following startle reaction to a pulse, has also been applied in the evaluation of tinnitus in animal models [65, 66], using PPI as a control experiment for preserved SMG. In the context of tinnitus, the carrier sound needs to be filtered to closely match the frequency range of the putative tinnitus, where an interference with startle inhibition is expected.

Numerous reports have identified a number of neural correlates of tinnitus in the dorsal cochlear nucleus of animals with GPIAS deficits [34, 40, 41], and have led to treatments such as direct vagus nerve stimulation or bimodal auditory/somatosensory stimulation with successful results in humans [3, 11, 12, 20, 44, 67], however using questionnaires as an outcome measure. Such research could benefit from objective quantification of treatment outcome. However, GPIAS is not as easily tested as PPI, requiring optimal sound environment and acoustics [43]. In mice, C57BL/6 mice show a robust GPIAS when gaps are presented close to the pulse (up to 80% suppression of the startle response) whereas CBA mice, that are more traditionally used for auditory studies because of their excellent hearing, have very poor GPIAS (near 20%) [70]. This led to a number of challenges to infer the presence of tinnitus in animal models [24], the macaque [52], and humans [23].

While the cochlear nucleus and inferior colliculus relay the primary auditory cues for both pre-pulses or gaps, the neural circuitry that is recruited during PPI or GPIAS differs. When exposing mice to repeated individual or combined PPI or GPIAS stimuli and assessing neuronal c-fos activation by immunohistochemistry in specific brain regions, inhibition by pre-pulses activated the lateral globus pallidus (LGP), and inhibition by gaps recruited the auditory cortex (AC) [45]. Indeed, lesions of the AC only impair the inhibition of the startle response by gaps, not by pre-pulses  [4]. Reversely, blockade of LGP activity with local injections of lidocaine impairs PPI [63], but whether it affects GPIAS remains unknown. Using optogenetics in the mouse auditory cortex, Weible et al. demonstrated that during GPIAS, inhibitory interneurons are mediating the comparison of pre- and post-gap spiking activity and modulating the degree of inhibition of the startle response [69].

Due to the strong reliance of the auditory cortex in modulating GPIAS, we hypothesized that sensory gating (SG), as measured by the cortical N1 evoked responses, could be used as a more reliable measure of inhibition by silent gaps when compared to SMG as measured by the blinking response. If so, this could become a new paradigm to be tested in the context of the objective assessment of tinnitus. As magnetoencephalography (MEG) primarily measures activity from the pyramidal neurons in the cortex [51], it appeared most suitable to test this hypothesis. We used source-localized MEG to provide a parametric high resolution spatiotemporal quantification of the inhibition of pulse-evoked N1 responses in presence of silent gaps in carrier BBN and simultaneously collect blinking responses for comparison. However, we opted for measuring blinking responses using electrooculograms (EOG), that are embedded in the MEG system, rather than using EMG. We investigated the critical parameters of pulse levels (70–95 dB), carrier BBN levels (60 and 70 dB), and inter-stimulus-intervals (ISI; 0, 60, 120, and 240 ms) to identify the optimal settings for cortical response inhibition and to compare it with blinking responses. We show a near 72.5% (SD ± 15.9) suppression of N1 90 dB pulse-evoked response when preceded 240 ms earlier by a 50 ms long silent gap in a 60 dB broadband carrier noise, and propose this approach for the objective assessment of auditory SG deficits as those that may occur in individuals with tinnitus.

**Table 1:** Descriptive characteristics of the participants. Between parenthesis are either standard deviation (SD) or percentage (%)

	**TinMEG (n=26)**
**Age (yr)**	
Mean (SD)	28.42 (5.81)
**Sex, n (%)**	
Male	12 (46.2%)
Female	13 (50.0%)
Other	1 (3.8%)
**Handedness, n (%)**	
Right	23 (88.5%)
Left	3 (11.5%)
**Tinnitus, n (%)**	
Yes, always	0 (0.0%)
Yes, often	0 (0.0%)
Yes, sometimes	0 (0.0%)
Not last year	9 (34.6%)
No never	16 (61.5%)
Don’t know	1 (3.8%)
**PSQ score**	
Mean (SD)	0.25 (0.16)
**HQ score**	
Mean (SD)	9.54 (6.11)
**HADS Anxiety score**	
Mean (SD)	4.65 (3.55)
**HADS Depression score**	
Mean (SD)	2.42 (3.14)
**PTA Left (dB HL)**	
Mean (SD)	2.70 (2.89)
**PTA Right (dB HL)**	
Mean (SD)	3.94 (3.02)
**HF PTA Left (dB HL)**	
Mean (SD)	6.57 (13.08)
**HF PTA Right (dB HL)**	
Mean (SD)	6.64 (10.01)
**LDL4 Left (dB HL)**	
Mean (SD)	88.58 (8.13)
**LDL4 Right (dB HL)**	
Mean (SD)	87.16 (7.74)

*PSQ* Perceived Stress Questionnaire; *HQ* Hyperacusis Questionnaire; *HADS* Hospital Anxiety and Depression Score; *PTA* Pure tone threshold average.125–8 kHz; *HF* High frequency 10–16 kHz; *LDL4* Loudness discomfort level at.5, 1, 2, 4 kHz; *dB HL* decibel hearing levels

## Methods

### Ethics Statement

The study was approved by the regional ethics committee in Stockholm, *Regionala etikprövningsnämnden* (Dnr:2019-05226). All subjects gave written informed consent after being informed about the aim and scope as well as risks of the study. The experiment was conducted in strict compliance with the ethical principles outlined in the Declaration of Helsinki.

### Study Population and Design

Demographics of the participants is shown in Table 1. This MEG study was designed to identify GPIAS parameters (interstimulus interval (ISI; 0, 60, 120, 240 ms), carrier (60 and 70 dB SPL), and pulse levels (from 70 to 95 dB SPL by steps of 5 dB)) that would achieve an optimal inhibition of the pulse response in presence of gaps, and compare the cortical evoked responses with the collected EOG. Normal hearing participants (*n*=26) were recruited via an online platform (accindi.se) for audiological assessment, MEG, and structural magnetic resonnance imaging (MRI). Exclusion criteria were pregnancy, sensitivity to sound (hyperacusis), psychiatric disorders, drug use, neurological disease, and non-removable metal implants. Sex was self-reported and well-balanced (13 females, 12 males and 1 other). Participants had a mean age of 28.4 (SD ± 5.8) and 88% of them had right handedness. Upon arrival at the national facility for magnetoencephalography (NatMEG) where the measures were performed in a single session, participants received detailed information on the procedure, signed an informed consent, confirmed they had no tinnitus, experienced no hearing difficulties, and filled in an on-line survey to assess stress, anxiety, depression and hyperacusis prior their auditory assessment (see section below). After successful MEG recording (total duration of 2.5–3 h), each participant was scheduled for a structural MRI scan (total duration 1 h) using a 3 Tesla GE MR750 Discovery at the MR Center, Karolinska Institutet. After completing both MEG and MRI sessions, all participants were compensated with 300 SEK for their time.

### Questionnaires and Audiological Measures

Swedish versions [46] of the Hyperacusis Questionnaire (HQ) [35], Perceived Stress Questionnaire (PSQ-30) [39], and the Hospital Anxiety and Depression Scale (HADS) [1] were delivered online (Sunet Artologik). Hearing thresholds were evaluated by high frequency Békésy-audiometry (0.125–16 kHz) with cut-offs for hearing loss defined at 25 dB HL following WHO guidelines (Astera2, Otometrics. Inc and HDA200 headphones, Sennheiser). Loudness discomfort levels (LDL) were tested in ascending steps of 5 dB at frequencies 0.5, 1, 2, and 4 kHz (Astera2, Otometrics. Inc and HDA200 headphones, Sennheiser).

### MEG Procedure

Before MEG scanning, all participants changed into MEG-compatible (cotton only) clothing provided and were instructed to remove all metal objects such as hair pins, piercings, and jewelry. Standard MEG preparation was performed including placement of position indicators (cHPI coils) to control for movement, 3D registration of the scalp (Polhemus) for co-registration with structural MRI. All MEG data was collected using a 306-channel Elekta Neuromag TRIUX system. As part of routine MEG procedures, EOG and electrocardiogram (ECG) were recorded and digitalized together with the MEG data. Indeed, measuring ECG is critical in MEG studies, since the magnetic component of heart beats introduce pronounced artifacts in MEG. ECG was collected from electrodes placed on the collarbones, which were later used to identify and correct artifacts with ICA (see MEG processing pipeline below). While EMG is traditionally used for measuring the direct muscle activity of the orbicularis oculi in startle studies in humans, vertical EOG can also help inferring on the magnitude of the blinking response [25]. Horizontal EOG, which is more frequently used to assess retinal activity, was not used in the blinking analysis. To minimize sleepiness during the exposure to BBN carriers, the participants were instructed to sit relaxed and watch a silent nature film on a screen with a display size of a 72 $\times$ 44 cm rectangle by an projector (FL35 LED DLP) situated outside the magnetically shielded room, while the different sound trials of the GPIAS paradigm were played. The MEG session concluded with a 5-min resting state recording in silence where the participants were instructed to sit relaxed, watching the movie as they had been for the previous part of the measurement. For the GPIAS paradigm, all sounds were presented pre-loaded from hardware (AudioFile stimulus processor; Cambridge research systems) through sound tubes (3.3 m, 12 mm and 4.5 mm wide) connected to one ADU1c acoustic driver unit (KAR audio) per ear.

### MRI Procedure and Analysis

A structural MRI was recorded on a separate day after having participated in the MEG session. The MRI data consist of 3D T1-weighted magnetization-prepared rapid gradient-echo (MPRAGE) sequence structural images (voxel size, 1 $\times$ 1 $\times$ 1 mm; field of view, 256 mm; RT, 2300 ms; ET, 2.98 ms) obtained on a GE Discovery 3.0T MR scanner. MRI data were processed with FreeSurfer (version 7) for cortical surface reconstruction and for creating source space models for MEG source reconstruction.

### GPIAS Paradigm

The critical parameters theorized to influence (cortical) inhibition by silent gaps were evaluated by modulations of, namely, carrier noise, pulse-level, and interstimulus interval (ISI). Trials were constructed with a uniform broadband white noise as carrier in MATLAB 2019b and exported as mono wav-files at 44.1 kHz sample rate. Level was calibrated using a sound level meter (Brüel & Kjær, 2235) coupled with a pre-amplifier (Brüel & Kjær, 2619) to an artificial external ear (Brüel & Kjær, 4157) at a level equivalent to fast, A-weighted dB SPL as measured for 15 s of continuous presentation - dB(A) SPL is hereafter referred to as dB for sake of brevity.

Two broadband carrier background of either 60 or 70 dB were used to present firstly increasing pulses (pulse only (PO); 20 ms with no rise and fall time) from 70 to 95 dB or 75 to 95 dB (with two ranges of relative intensities: +10 to +35 dB and +5 to +25 dB, respectively), secondly with silent gaps (duration of 50 ms with a 2 ms sinusoidal rise and fall ramp) in absence (gap only (GO)) or in presence of a 90 dB pulse (gap pulse (GP), to avoid exposure to louder sound intensities), using ISIs of 0, 60, 120, or 240 ms. In one 5-min block, all trials were presented 5 times each in random order with a 2-s interval (jittered by 0.5 sec), first in a 60 dB carrier white noise, followed by presentation in a carrier noise of 70 dB.

Trial randomization and inter-trial jitter, as well as synchronization of trigger channels, were performed by a custom script in Presentation software (Version 18.0, Neurobehavioral Systems, Inc.). After each block, we communicated with the participant via an intercom system to make sure they were comfortable and assessed sleepiness using the Karolinska Sleepiness Scale (KSS) [55]. Ten blocks were repeated for a total of 50 presentations of each trial type, and the total scanning time was around 90 min.

Of note, this study did not include any PPI experiments, since measurements from structures of the basal ganglia such as the LGP cannot be obtained using MEG. Neurons in the LGP are not neatly aligned in parallel as the synapses of the pyramidal neurons in cortex are. As a consequence, we assumed there would not be enough summation of the electric/magnetic fields to generate a strong enough signal to be measured outside the head. Would the anatomy not have been a hindrance, the LGP is too distant from the MEG sensors outside the head to be picked up, as the signal decay of the magnetic field over distance is the inverse cubed power.

### MEG Processing Pipeline

The MEG data was recorded with a sampling rate of 5000 Hz. We generated signal-space projection (SSP) vectors [68] from both gradiometer and magnetometer sensors, using two empty-room recordings taken on the same day as the subject’s experiment, before and after the experiment. These SSP vectors were then employed to eliminate environmental noise originating from sources outside the subject’s body and the MEG system in the data. Next, we utilized the signals from continuous head position indicator (cHPI) coils to track the subject’s head position over time, which is used for subsequent compensation in the recordings. Afterwards, we automatically identified and corrected noisy and flat MEG channels, as well as crosstalk compensation among the well-functioning MEG channels [64]. We concluded this by performing spatiotemporal signal-space separation (tSSS) on the data in 10-s chunks for computing temporal projections [30, 64]. Subsequently, the data was downsampled to 250 Hz. Prior to downsampling to prevent aliasing, a low-pass filter with a cutoff frequency of 500 Hz was applied to the recordings. The MEG recordings were then bandpass filtered within the range of 0.1 to 40 Hz.

The data was further broken down into independent components (ICs) for the purpose of artifact correction, utilizing the Infomax method [2]. We determined the minimum number of components necessary to collectively account for at least 95% of the data’s variance. Specifically for the ECG channel, we applied a bandpass filter between 8 and 16 Hz and identified the relevant ICA component, which was subsequently removed using the cross-trial phase statistics method [13]. In the case of muscle artifacts, we used the approach detailed in Dharmaprani et al. [16] to identify and remove any associated ICA components. Notably, ICA components representing vertical eye movements were retained in the data. This decision was made because not all the activities detected by ICA were actual blinks; some were ocular (startle) responses to the auditory stimuli.

Consequently, after epoching the data, we established a custom threshold (500 $\mu v$) to selectively remove epochs with blinks exceeding the threshold, effectively eliminating “voluntary” blinks while preserving ocular responses to the auditory stimuli. This is based on evidence showing that voluntary blinks exhibit a significantly greater amplitude compared to spontaneous (involuntary) blinks [14]. The recordings are segmented into epochs, covering a time window of 300 ms before the stimulus onset to 300 ms after the stimulus onset. The period ranging from 300 ms before the stimulus until the stimulus onset is designated for baseline correction. Epochs exhibiting a peak-to-peak signal amplitude less than 1 femtotesla or exceeding 4 picotesla (for magnetometers) are discarded. Similarly, epochs with a peak-to-peak signal amplitude below 1 fT/cm or exceeding 400 pT/cm (for gradiometers) are also excluded from subsequent processing.

We performed the anatomical cortical surface reconstructions of the MRIs using FreeSurfer software [21]. Following that, we generated boundary element model (BEM) surfaces, including the inner skull, outer skull, and outer skin (scalp), utilizing the watershed algorithm [30, 54]. We established a surface-based source space with bilateral hemisphere representation, recursively dividing it with octahedron spacing. For each subject, we constructed a BEM model and its corresponding solution using the linear collocation method [30]. To ensure proper alignment, we performed co-registration between the MRI and the head model, utilizing three fiducial points provided in the MRI as an initial solution. This transformation was further refined through 40 iterations of the iterative closest point (ICP) algorithm [10]. Any outlier points exceeding a distance of 5 cm were excluded, and the fitting process was repeated. For each subject, we computed the forward solution using the source model, BEM model, and co-registration information. We calculated the noise covariance of the recordings, focusing on the pre-stimulus periods of the pulse-only stimuli, as these periods are devoid of event-related brain activity. We used both the *empirical* [30] and *shrunk* [38] methods to estimate the noise covariance and selected the best estimator based on log-likelihood and cross-validation with unseen data [19]. Using the noise covariance and the forward solution, we applied the linear minimum-norm inverse method, known as *dSPM* to determine the inverse solution [30]. This enabled us to obtain source time courses for each vertex in the source space. To facilitate group-level analysis and ensure that spatial locations could be compared uniformly across all subjects, we morphed each individual’s source space onto a template brain. This involved a linear mapping of cortical surface values from each individual subject to those in a FreeSurfer template brain [21]. Next, we generated a single time course for each brain label by averaging the source time courses within the vertices located within that specific brain label, regardless of their orientation. The brain labels and cortical parcellation were derived from the Desikan-Killiany Atlas [15] with 68 bi-hemispheric parcels.

Further analysis steps included dividing the continuous recording in epochs ± 600 ms relative each pulse trigger onset for each separate stimuli type. The data was then inspected for each stimuli type, and high variance trials were manually excluded for both gradiometer and magnetometer channels. All trials were compiled, and ECG channel was used to identify components of the heartbeat that could create artifacts. These were removed from the full dataset using independent component analysis (ICA) in a standard pipeline. The data was not processed to remove ICA components, corresponding to vertical eye movements from the EOG channels as high correlation between eye blinks and any response of interest related to the pulse stimuli was expected. The EOG channel, which records vertical eye movements to detect blinks, was analyzed independently to compare the traditional muscle reflex with cortical responses. The EOG channel, which captures a mixture of eyelid movements (blinks) and vertical eye movements, was analyzed independently to compare cortical responses with peripheral signals related to blink-like activity. EOG signals were recorded using the standard NatMEG electrode placement setup, as detailed at the following link. The vertical EOG electrode was positioned above the eye to capture signals related primarily to eyelid movements, particularly blinks, though contributions from vertical eye movements cannot be entirely ruled out. Signals were digitized at 250 Hz and bandpass filtered between 1 and 10 Hz using a Hanning-windowed finite impulse response (FIR) filter with a 0.5 Hz transition bandwidth. This filter was applied bidirectionally to ensure zero-phase distortion. The selected frequency range was chosen to isolate blink-related transients from slower ocular movements. Blinks were automatically detected using a peak detection algorithm, with the detection threshold defined as one-fourth of the EOG signal’s amplitude range: $(max(EOG) - min(EOG)) / 4$. EOG responses surpassing a certain threshold (i.e., 500 $\mu v$) were assigned as spontaneous blinks, resulting in the removal of such trials. Data from individual gradiometer sensors and those with the highest event-related field (ERF) amplitude among most participants were examined separately on the left and right sides of the array. All MEG processing analyses were performed using MNE software (Version 1.6.1).

### Analysis and Statistics

Responses for all trials (i.e., EOG, ERP peak latency an amplitude) were analyzed in a defined time window of interest of 85–115 ms with a two-way analysis of variance (ANOVA). To identify a response as a blinker, we used a thresholding response whereby an EOG signal exceeding the average EOG signal before the stimulus onset value + 10 * the median absolute deviation of the same pre-stimulus period was considered a blink. The percentage of inhibition for the four different ISIs were calculated as [1-(GP/PO)], where GP represent area under curve in trials with a gap preceding the pulse and PO trials with a pulse only. The resulting values denoting the percentage of inhibition were then also subjected to two-way ANOVA. For the individual contrasts between conditions, post hoc tests and *P*-values reported are corrected for multiple comparisons using the Tukey method. All statistical analyses have been performed with Python (Version 3.10.9) and the packages SciPy (Version 1.13.0). Statistical test assumptions were checked before testing and the global two-tailed significance level was set to p< 0.05.

**Fig. 1:**
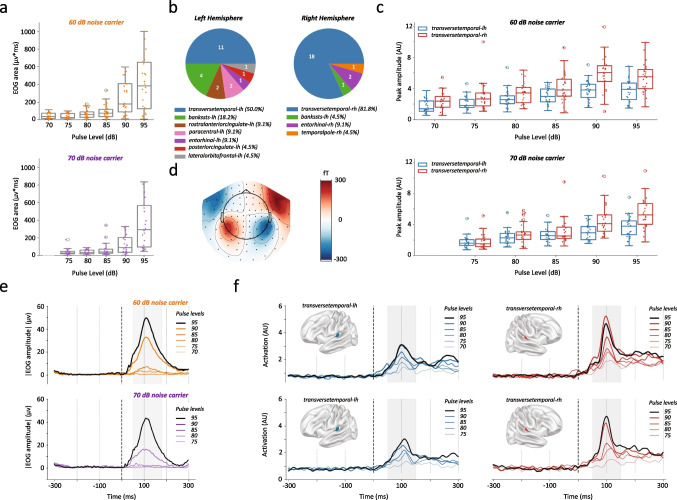
Blinking and cortical responses to increasing pulse intensities. **a** Distribution of EOG area under curve values for multiple pulse levels starting from 70 to 95 dB with 60 dB (upper panel, orange) and 70 dB (bottom panel, purple) broadband noise carrier. **b** Pie plots representing the number of subjects with maximal activity in left hemisphere (left panel) and right hemisphere (right panel) brain parcels. **c** Distribution of peak amplitude values in the left (blue) and right (red) transverse temporal gyrus for multiple pulse levels within 70 and 95 dB; upper and lower panels display peak amplitude values with 60 and 70 dB broadband noise carrier level, respectively. **d** Grand averaged topographic distribution in the canonical N1-time window in response to 90 dB pulse stimulus. **e** Average EOG responses for pulse levels between 70 and 95dB in steps of 5 dB with 60 dB (upper panel, orange) and 70 dB (bottom panel, purple) broadband noise carrier. Highest intensity responses (95 dB) are colored in black. **f** Average source level analysis of left and right transverse temporal gyrus show an expected increase of activation with rising pulse level in both 60 (upper panels) and 70 dB (bottom panels) broadband carrier noise for both left (blue lines) and right (red lines) transverse temporal gyrus. Box plots show individual values together with median ± 1.5 x IQR (inter quartile range), *n* = 22–26

## Results

### Cortical Responses Are More Sensitive than Blinking Responses to Sound Pulses of Increasing Intensity

We first determined the amplitude of EOG and ERP responses to increasing intensities of sound pulses in two different broad band carrier noises (BBN) of 60 and 70 dB. As expected, startle responses from the eye blinking were observed at lower sound pulses with the lower carrier level (60 dB, BBN) starting being evident at 90 dB and reaching a maximum average amplitude of 64.3 $\mu$V ± SD 75.9 with 95 dB pulses (two-way ANOVA, pulse level factor: *F*(4, 200) = 16.935, *P* = 4.74e$-$12, Fig. 1a, Tables 2 and 3). With higher carrier level (70 dB, BBN), the average EOG amplitude in response to a 95 dB pulse was 58 $\mu$V ± SD 62.0. The EOG responses to increasing stimulus levels did not differ between 60 and 70 BBN carrier levels (two-way ANOVA, carrier level factor: *F*(1, 200) = 1.378, *P* = 2.41e$-$1, Table 2).

The same was found for ERPs, where source reconstruction using individual’s MRI scans shows that the 82.6% of the peak signals dominantly appeared in the transverse temporal region in the right hemisphere and 47.8% in the left hemisphere (Fig. 1b). Nonetheless, these were more linear in their progression over pulse levels than EOG and becoming evident at 90 dB (two-way ANOVA, LH pulse level factor: *F*(4, 200) = 17.06, *P* = 3.94e$-$12, RH Carrier level factor: *F*(4, 200) = 31.86, *P* = 9.49e$-$21, Fig. 1c, Table 2 and 3). Signals were greater in a 60 dB BBN carrier when compared to the 70 dB one (two-way ANOVA, LH carrier level factor: *F*(1, 200) = 6.99, *P* = 0.009; RH carrier level factor: *F*(1, 200) = 16.96, *P* = 5.49e$-$5, Table 2). The ERPs were predominant on the right hemisphere, being $\sim$ 1.5-fold greater than on the left hemisphere in both carrier levels. Topoplots of ERPs measured at 100 ms confirm a typical auditory response signal with dipoles located symmetrically over the two ears (Fig. 1d). Averaged traces for the EOG and ERPs in response to pulses in both 60 and 70 dB carrier levels are illustrated in Fig. 1e–h. Similar trends for the ERPs were found at the sensor level Supplementary Fig. 1. Noteworthy, we used a thresholding method to determine the percentage of blinkers in response to specific pulse intensities. In the 60 dB carrier noise, a 75 dB pulse triggered blinks in 9% of the participants, and reaching 68% of blinkers at 95 dB pulses.

The latency of the ERPs consistently peaked at 100 ms across all pulse levels (two-way ANOVA, LH pulse level factor: *F*(4, 84) = 1.12, *P* = 0.348; RH pulse level factor: *F*(4, 84) = 0.56, *P* = 0.691, Table 2). This absence of differences was expected given the experimental design with identical, temporally fixed, short, and basic auditory pulse stimuli. In contrast, EOG latencies were less sensitive to pulses of lower intensities, reaching 202.6 ms ± SD 95.0 and 218.7 ± SD 91.0, in a 60 or 70 dB BBN carrier, respectively (two-way ANOVA, EOG pulse level factor: *F*(4, 84) = 10.32, *P* = 1.19e$-$7; EOG carrier level factor: *F*(1, 21) = 0.24, *P* = 0.618, Table 2).

### Silent Gaps Trigger Cortical Responses but Not Blinking Responses

**Fig. 2:**
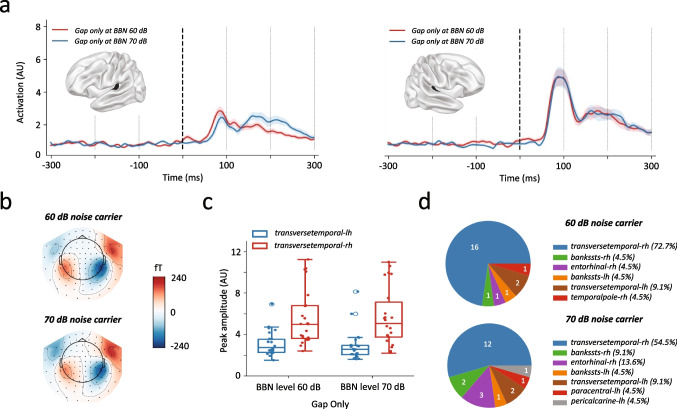
Silent gaps trigger cortical responses but not blinking responses. **a** Averaged activation in right transverse temporal gyrus for gap only stimuli with 60 (red line) and 70 dB (blue line) background noise carrier. Shadowed lines represent the standard error of the mean. **b** Grand averaged topographic distribution in the canonical N1-time window in response to gap only stimuli background noise level of 60 (upper panel) and 70 dB (bottom panel). **c** Distribution of maximum activation in left (blue) and right (red) transverse temporal gyrus in two different background noise carrier (60 and 70 dB). **d** Pie plots representing the number of subjects with maximal activity in brain labels in two different background noise level including 60 dB (upper panel) and 70 dB (bottom panel). Box plots show individual values together with median ± 1.5 x IQR. *n* = 22-26

We next evaluated the responses to GO, that is in absence of pulses, in a 60 or 70 dB broad band carrier noise. Whereas gaps did not trigger any blinking response, they did elicit positive deflections in the cortex, which were bigger in the right hemisphere (one-way ANOVA with *F*(1, 21) = 15.74, *P* = 0.00027 for 60 dB and *F*(1, 21) = 16.69, *P* = 0.00019 for 70 dB BBN, Fig. 2a–c, Table 4), with transverse temporal being the main recruited region dominating in 73.9% of peak signals in the right hemisphere versus 56.5% in the left hemisphere (Fig. 2d). However, unlike the pulse response, which consists mainly in ipsilateral responses, GO elicited a response biased to the right transverse temporal region.

### Effective Inhibition of Cortical Responses to Pulses in Presence of Silent Gaps

**Fig. 3:**
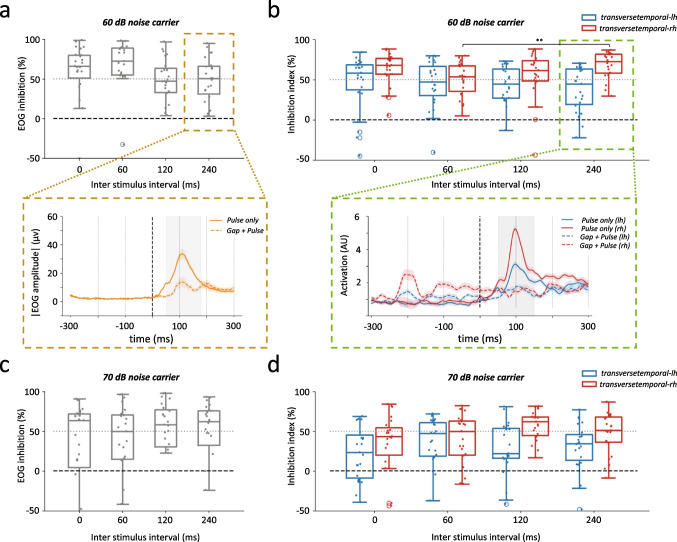
Effective inhibition of cortical responses to pulses in presence of silent gaps. Distribution of the EOG inhibition index values (**a**) and inhibition index values computed in left and right transverse temporal gyrus (**b**) in the 60 dB broadband noise carrier level, using the 90 dB pulse as the baseline condition. Each dot represents the ratio between the area under the curve of the responses in presence or absence of a gap for various interstimulus intervals (0, 60, 120, 240 ms). EOG and ERP inhibition index values in a 70 dB carrier BBN carrier are shown in **c** and **d**, respectively. Magnified in the middle panel box are the grand averaged EOG response (orange) and the activation in left (blue) and right (red) transverse temporal gyrus at 60 dB BBN level for the pulse only (continuous line) and gaps followed 240 ms later by pulses (dashed line). Box plots show individual values together with median ± 1.5 x IQR, *n* = 22–26. $$^{**}  \textit{p} < 0.05$$ (i.e., the difference between 60 and 240 ms at 60 dB BBN at the right transverse temporal gyrus). Non-significant simple effects are not shown here for sake of clarity

With 95 dB and 90 dB pulses being of very similar efficacy in triggering EOG and ERPs (Table 3 and Supplementary Table. 1), we selected the latter, being more comfortable for the participants due to its lower loudness. We next quantified the degree of inhibition (%) of the EOG and ERPs in response to 90 dB pulses by comparing the amplitudes in absence or presence of a gap when presented at different interstimulus intervals (ISI). A value of 100% reflects a complete suppression of the response to pulses, whereas 0% indicates no inhibition has happened. Unlike what has been shown in rodents where the degree of inhibition decreases with more distal gaps [70], here, gaps displayed similar efficacy in suppressing the startle whether at 240 ms or 0 ms ISI whether measured with EOG or ERPs (Fig. 3), with the exception of the 240 ms gaps in the right hemisphere reaching 72.48% ± SD 15.9 in a 60 dB BBN carrier (two-way ANOVA, ISI factor: *F*(3, 63) = 2.80, *P* = 0.042; Fig. 3, Table 5). Cortical inhibition in both left and right hemisphere was sensitive to the carrier level (two-way ANOVA, left hemisphere carrier level factor: *F*(1, 21) = 10.08, *P* = 0.002; right hemisphere carrier level factor: *F*(1, 21) = 12.71, *P* = 0.0005, Table 5), whereas this was not the case of the blinking responses (two-way ANOVA, EOG carrier level factor: *F*(1) = 3.73, *P* = 0.055, Table 5). The degree of inhibition across various ISI for the 70 dB BBN carrier is shown in Supplementary Fig. 2. The maximum EOG inhibition occurred at 60 ms ISI in a 60 dB BBN carrier (72.5% ± SD 37.81), but not differing from other ISIs (two-way ANOVA, EOG ISI factor: *F*(3, 63) = 0.43, *P* = 0.729, Table 5).

**Table 2:** Mean amplitudes and peak latency (in milliseconds) of the electrooculograms (EOG) and cortical ERPs in both left and right brain hemispheres (LH and RH) in response to varying pulse levels (20 ms in duration, from 70 to 95 dB) presented in either a 60 or 70 dB carrier broad band noise. EOG peak amplitudes are expressed in ($\mu$V), while cortical responses are measured in arbitrary unit (AU). Data are mean (±SD), and refer to Fig. 1. Two-way ANOVA are reported. Significant *P* values $<0.05$ are marked in bold

	**Peak amplitude**					
	**60 dB**			**70 dB**		
Pulse Level (dB)	EOG	LH	RH	EOG	LH	RH
70	6.6 (4.3)	1.8 (0.9)	2.5 (1.2)			
75	7.0 (3.6)	2.2 (0.9)	3.0 (1.9)	7.3 (5.1)	1.8 (0.8)	1.9 (1.0)
80	10.7 (6.7)	2.8 (1.1)	3.6 (1.3)	6.3 (5.0)	2.4 (1.0)	3.0 (1.4)
85	13.7 (11.1)	3.1 (1.0)	4.5 (1.7)	9.9 (9.0)	2.6 (1.0)	3.2 (1.7)
90	42.4 (42.3)	3.8 (1.3)	6.4 (2.0)	26.8 (43.2)	3.1 (1.1)	4.8 (1.9)
95	64.3 (75.9)	3.8 (1.3)	5.7 (1.7)	58.0 (62.0)	3.8 (1.4)	5.8 (1.8)
	Two way ANOVA					
	EOG		LH		RH	
Pulse level (dB)	*F*(4, 84) = 16.93; *P* = **4.74e–12**		*F*(4, 84) = 17.06; *P* = **3.94e–12**		*F*(4, 84) = 31.86; *P* = **9.49e–21**	
Noise carrier level	*F*(1, 21) = 1.38; *P* = 0.2416		*F*(1, 21) = 6.99; *P* = **0**.**0087**		*F*(1, 21) = 16.96; *P* = **5.49e–05**	
Interaction	*F*(4, 84) = 0.25; *P* = 0.9091		*F*(4, 84) = 0.36; *P* = 0.8369		*F*(4, 84) = 1.66; *P* = 0.1609	
	**Peak latency (ms)**					
	**60 dB**			**70 dB**		
Pulse level (dB)	EOG	LH	RH	EOG	LH	RH
70	181.5 (107.6)	102.0 (7.8)	100.5 (6.5)			
75	202.6 (95.0)	100.9 (6.9)	98.1 (6.4)	218.7 (91.0)	102.5 (6.7)	101.5 (7.5)
80	135.6 (77.9)	100.0 (5.3)	101.8 (6.3)	188.7 (104.8)	100.0 (7.5)	100.6 (7.3)
85	134.3 (72.1)	101.6 (5.8)	99.5 (6.4)	136.0 (94.8)	98.6 (7.7)	101.8 (7.7)
90	123.5 (66.0)	99.9 (6.6)	98.7 (4.6)	111.0 (73.4)	99.3 (7.1)	99.3 (5.5)
95	110.4 (41.5)	99.8 (5.5)	100.4 (4.9)	109.5 (30.6)	100.1 (5.5)	100.2 (5.0)
	Two way ANOVA					
	EOG		LH		RH	
Pulse level (dB)	*F*(4, 84) = 10.32; *P* = **1.19e–07**		*F*(4, 84) = 1.11; *P* = 0.3481		*F*(4, 84) = 0.56; *P* = 0.6907	
Noise carrier level	*F*(1, 21) = 0.24; *P* = 0.6181		*F*(1, 21) = 1.05; *P* = 0.3071		*F*(1, 21) = 1.58; *P* = 0.2091	
Interaction	*F*(4, 84) = 0.84; *P* = 0.4960		*F*(4, 84) = 0.93; *P* = 0.4454		*F*(4, 84) = 1.29; *P* = 0.2722

**Table 3:** Mean amplitudes and peak latency (in milliseconds) of the cortical ERPs in both left and right brain hemispheres (LH and RH) in response to silent gaps (50 ms in duration) presented in either a 60 or 70 dB carrier broad band noise. Cortical responses are measured in arbitrary unit (AU). Data are mean (± SD) and refer to Fig. 2

**Peak amplitude**					
**60 dB**			**70 dB**		
EOG	LH	RH	EOG	LH	RH
7.9 (6.6)	3.2 (1.2)	5.7 (2.7)	8.6 (6.2)	2.9 (1.5)	5.7 (2.8)
One way ANOVA					
EOG		LH		RH	
*F*(1, 21) = 0.13; *P* = 0.7108		*F*(1, 21) = 0.32; *P* = 0.5734		*F*(1, 21) = 0.03; *P* = 0.9545	
**Peak latency (ms)**					
**60 dB**			**70 dB**		
EOG	LH	RH	EOG	LH	RH
200.9 (85.9)	100.5 (6.4)	99.4 (4.6)	173.8 (99.2)	103.4 (6.4)	100.0 (4.4)
One way ANOVA					
EOG		LH		RH	
*F*(1, 21) = 0.94; *P* = 0.3385		*F*(1, 21) = 2.20; *P* = 0.1451		*F*(1, 21) = 0.25; *P* = 0.6174	

**Table 4:** Degree of inhibition of the blinking response and cortical inhibition in response to a 90 dB pulse presented in presence or absence of a silent gap in either a 60 or 70 dB BBN carrier. Values expressed in % reduction of the response in presence of a gap when compared to a response without a gap. Gaps are presented at different interstimulus intervals (ISI) of 0, 6, 120, and 240 ms. The electrooculogram (EOG) inhibition and the cortical inhibition index values are provided for both the left and right transverse temporal gyrus (LH and RH). Results from a Two way ANOVA are reported. Data are mean (± SD) and refer to Fig. 3. Significant *P* values $<0.05$ are marked in bold

	Inhibition index (%) in the 60 dB noise carrier		
	EOG	LH	RH
ISI 0ms	66.35 (43.26)	58.64 (34.77)	68.31 (20.42)
ISI 60ms	72.50 (37.81)	47.19 (29.6)	53.70 (20.69)
ISI 120ms	47.19 (35.56)	45.00 (22.26)	61.68 (30.51)
ISI 240ms	50.64 (41.08)	44.92 (27.24)	72.48 (15.88)
	Inhibition index (%) in the 70 dB noise carrier		
	EOG	LH	RH
ISI 0ms	63.89 (162.14)	23.46 (33.54)	43.23 (46.19)
ISI 60ms	49.89 (549.19)	47.43 (46.58)	50.21 (61.44)
ISI 120ms	58.31 (814.15)	22.14 (33.49)	62.10 (32.74)
ISI 240ms	62.56 (1589.82)	34.32 (30.35)	51.2 (25.51)
	Two way ANOVA		
	EOG	LH	RH
Noise carrier level	*F*(1, 21) = 3.73; *P* = 0.0552	*F*(1, 21) = 10.08; *P* = **0**.**0017**	*F*(1, 21) = 12.71; *P* = **0**.**0005**
ISI	*F*(3, 63) = 0.43; *P* = 0.7286	*F*(3, 63) = 0.09; *P* = 0.9605	*F*(3, 63) = 2.80; *P* = **0**.**0416**
Interaction	*F*(3, 63) = 0.35; *P* = 0.7867	*F*(3, 63) = 0.46; *P* = 0.7077	*F*(3, 63) = 1.45; *P* = 0.2298

**Table 5:** Results of the Tukey HSD (Honestly Significant Difference) multi-comparison test from comparisons within broad band noise (BBN) carrier noise levels and across different interstimulus intervals of 0, 60, 120, and 240 ms from Supplementary Table 4. These results refer to Fig. 3. Significant *P* values $<0.05$ are marked in bold

Multiple comparison of means (background noise level)					
Group 1	Group 2	Mean diff	*p*-adjusted	Lower	Upper
60	70	$-$18.4701	**0**.**0006**	$-$28.8932	$-$8.0469
Multiple comparison of means (inter stimulus interval)					
Group 1	Group 2	Mean diff	*p*-adjusted	Lower	Upper
0	120	5.6124	0.8815	$-$14.1064	25.3311
0	240	9.6306	0.5851	$-$10.0881	29.3493
0	60	$-$10.3609	0.5242	$-$30.0796	9.3578
120	240	4.0182	0.952	$-$15.7005	23.7369
120	60	$-$15.9733	0.1567	$-$35.692	3.7454
240	60	$-$19.9915	**0**.**0456**	$-$39.7102	$-$0.2728

The EOG responses appeared more variable than ERPs, and more so in the 70 dB BBN carrier, reaching a standard deviation 25 times greater than the mean in the 240 ms ISI. To ascertain if there is a relation between elicited blinks and cortical responses, we probed the correlation between EOG and ERPs in response to the 90 dB pulse, and the correlation in the inhibition index in the 60 dB BBN carrier. We found no correlation between the blinking amplitudes and the cortical responses, meaning that stronger EOG did not relate to larger cortical ERPs. Likewise, no correlation was found between EOG responses and the inhibition index (Supplementary Fig. 3). Overall, the absence of these correlations suggests a missing or unrelated EOG response, or that the induced EOG motor output was subjected to greater variability than the cortical responses. Taken together, we identified the 90 dB pulse, in a 60 dB carrier with gaps presented at 240 ms ISI as the optimal parameter to induce cortical inhibition.

## Discussion

Our findings show that a GPIAS paradigm tested using source reconstructed MEG is able to accurately measure cortical inhibition of the N1 response to a sound pulse when preceded 240 ms before by a 50 ms long silent gap. An ISI of 240 ms was found optimal for maximum cortical inhibition and for separating response components related to the on- and off-set of the gap from those related to the following pulse. We propose this paradigm as a new means of investigating inhibition mechanisms occurring during auditory SG in humans, to be tested for instance during the development of language and during aging, and potentially in the objective diagnosis of tinnitus.

After evidencing that temporal processing is impaired in patients with schizophrenia, PPI has become an influential experimental paradigm in neuropsychophysiology in animal and humans [7]. Its use in pre-clinical models has helped evidencing the primary role of dopamine in the ventral striatum as the main neurochemical and anatomical substrates involved in PPI [57, 59], further extending to the forebrain and pontine circuit [58]. PPI also further helped understanding the developmental and genetic contributions to SMG deficits [26, 27, 42], which has led to the establishment of models for antipsychotic discovery. In contrast, the “subjective” detection of gaps in sounds (or their discrimination)—thought to be analogous to speech events such as voice-onset time for consonants—helped inferring on the temporal processing occurring during speech perception, a phenomenon that is reduced in hearing impaired individuals, even when corrected for sensation levels [22]. However, the ability of gaps to suppress the startle response was introduced much later with the purpose of objectively detecting tinnitus in animals [65, 66]. Because lesions of the auditory cortex impair GPIAS but not PPI [4, 33], and that GPIAS and PPI recruit different neural pathways [45], both paradigms assess different aspects of SMG.

In humans, silent gaps can also inhibit the blinking reflex to a degree similar to PPI [23, 49]. This inhibition has been reported to increase with gap duration, as well as ISIs up to 100 ms, and increasing carrier levels [37], which contrasts our findings whereby the inhibition of EOG responses was found equally effective from 0 to 240 ms, whatever the carrier level was. We note that inhibition measures EOG were very variable (CV = 1590%) impacting the statistical power of this measure, when compared to the measures of inhibition using cortical event-related fields (CV = 25.5%). Such variability could also be grounds to the difficulty of achieving good inhibition in PPI studies in humans, where there is poor control over background noise intensities (e.g., not being performed in a sound-proof room or with in-ear inserts) requiring a high carrier level (e.g., 70 dB) [62]. Our findings suggest that the GPIAS paradigm could be reliably tested using cortical responses, rather than blinking responses. Whether an equivalent sensitivity to assess gap-mediated inhibition can be achieved with EEG as compared to MEG will require thorough testing as EEG systems vary in their sensitivity depending on the hardware and processing systems. This will be critical for a wide-spread clinical use of this paradigm.

Our findings are also in support of GPIAS being an automatic, pre-conscious, measure of inhibition. According to the “tinnitus fills the gap” hypothesis, which is based on the subjective gap detection paradigm used in auditory gating studies, impaired startle inhibition in tinnitus individuals when the carrier frequency closely matches that of the perceived tinnitus would occur due to the fact that gaps would no longer be perceived. Studies in mice and humans demonstrated that silent gaps can still be subjectively perceived in the context of tinnitus [5, 8]. Instead, we believe that the inhibition of this motor reflex response is strongly regulated in the auditory cortex, where ERPs to a 90 dB pulse can be almost completely suppressed by gaps, preceding in time any subjective detection of the gap. Optogenetic studies in mice demonstrated that inhibitory interneurons in the auditory cortex process pre- and post-gap spiking activity to regulate the inhibition or the enhancement of the startle response [69]. Epicranial studies in awake mice have successfully measured inhibition to pre-pulses in the region of the auditory cortex [36]. The back-translation and adaptation of the present paradigm to such pre-clinical procedures may help further understanding the fundamental mechanisms underlying gap-mediated inhibition of auditory cortex activity.

Auditory ERPs have been shown to differ between left and right hemispheres with bilateral stimulation in experiments of mismatch negativity [28, 32, 53] and to contribute to the N1 response components differently for gaps in noise [50]. Our analysis highlights the importance of considering the responses in the right and left hemispheres, as right hemisphere response amplitudes were 1.5-fold larger for both gaps and pulses, and thus a greater dynamic range for assessing an impact on inhibitory mechanisms. Of note, bi-hemispheric separation in auditory ERP analysis in MEG is a common practice given the perpendicular nature of magnetic fields vs. electrical dipoles of EEG leading to ERPs in the center of the scalp topography. Overall, our lateralized findings are in line with common observations regarding auditory ERPs in MEG  [53] and possibly reflective of (auditory) attentional effects usually lateralized to the right hemisphere [17, 56]. Focusing specifically on auditory stimuli and attention, the right hemisphere has a unique role in processing and directing attention to these stimuli. It is involved in distinguishing and responding to complex auditory information, especially when it involves emotional or spatial elements. This lateralization can influence how we perceive and react to sounds, including the ability to focus on specific auditory inputs in environments filled with multiple stimuli. To further elucidate whether the right hemisphere’s superior ability to register the gap is what drives the inhibition of the response in both hemispheres will require future studies with alternating monaural presentation of silent gaps and following pulses.

The present study has a number of limitations. First, we emphasize that we have used EOG, instead of EMG traditionally used during SMG studies. This was due to the standardized use of EOG during MEG measures. Indeed, EMG is more sensitive than EOG [25], since EMG can be measured even when the response is too weak to move the eyelids. In addition, EOG does not exclusively measure blinking responses, since it can also be affected by rotation of the eyeball (causing false positives). EOG can also be due to either contraction of orbicularis oculi or relaxation of levator palpebrae, both of which will result in lid descent. With regard to blinks, there are mainly three types: spontaneous, voluntary, and reflexive blinks. Spontaneous blinks, in terms of amplitude, fall within the range of reflexive responses to pulses from the smallest to the largest pulse intensities (Supplementary Fig. 4a). In contrast, a problematic with too strong EOG signals is that they become important confounders in the MEG measures. Indeed, the MNE toolkit recommends exclusion of vertical EOG signals greater than 250 $\mu v$. We thus employed a pragmatic approach by excluding voluntary blinks with responses larger than 500 $\mu v$ captured on vertical eye movements. In addition to the exclusion of horizontal eye movements, we aimed using this approach, to minimize the confounding effects of these unspecific EOG signals, while still being able to measure reflex blinking responses to sound pulses and compare them to the N1 evoked responses. This lead to near 10% of trials being removed because of too large EOG signals, however ensuring a greater quality of data (Supplementary Fig. 4b). Since these exclusions were consistent across all trial types (Supplementary Fig. 4c), we believe this did not negatively impact the outcome. Second, the adaptation of the GPIAS paradigm to cortical evoked responses was performed on healthy normal hearing individuals without tinnitus. For it to be a reliable and sensitive diagnostic tool for tinnitus, it remains to be assessed how the N1 pulse response, and its inhibition by silent gaps, can be affected by age, sex, hearing loss at low or high frequencies, stress and/or anxiety, and hyperacusis, some of which are strong confounders in SMG [61] and in tinnitus [9, 18]. With a larger sample size, the impact of these factors could have eventually been addressed. Second, ethnicity or cultural background is known to significantly impact SMG as evidenced using EMG-PPI [60]. Therefore, as the present study was only performed on participants living in the Stockholm area, the universality of our MEG findings will have to be tested on other ethnicities. Finally, MEG data could have been collected simultaneously to EEG in order to compare their relative sensitivity. As MEG is less amenable to clinical routine, such comparison may allow the adaptation of this paradigm to the bedside. All of these aspects will need to be evaluated in the future to ensure a broad applicability of this paradigm in the auditory field.

## Conclusion

The findings that cortical inhibition can be effectively achieved using a GPIAS paradigm establish a framework for testing the development of auditory SG in children and the elderly, as well in the context of diagnosis of disorders such as tinnitus, for which the GPIAS was initially intended [23, 24]. For the latter, careful questionnaire and auditory phenotyping should be performed to minimize the heterogeneity of the tested tinnitus group, which could potentially mask positive associations. Indeed, recent studies have shown that hyperacusis is a strong confounder in tinnitus [9], that could mask the identification of tinnitus electrophysiological signatures as shown in auditory brainstem response studies [18, 31]. Should the cortical GPIAS find successful relationships with a tinnitus phenotype, this may establish a basis for strong translationally valid paradigm to better diagnose and understand the fundamental mechanisms underlying this condition.

## Supplementary Information

Below is the link to the electronic supplementary material.

Supplementary file 1 (pdf 1105 KB)

## Data Availability

Large raw data files, including electrophysiological, video, or imaging data, are available upon request from the corresponding author. As data is not de-identified, data access will require a data processing agreement and ethics approval.

## Code Availability

The codes used for presenting GPIAS paradigm, analyzing MEG and MRI data and generating figures, are available in the following repository (https://github.com/translational-audiology-lab/TinMEG).
