# Supplementary material for: Inhibition of Cortical Evoked Responses to Sound Pulses by Preceding Silent Gaps
Source: J Assoc Res Otolaryngol. 2025 Jul 23;26(5):515–29. doi: 10.1007/s10162-025-00999-w (PMC12528562; doi:10.1007/s10162-025-00999-w)
Supplement: Supplementary file 1 — (pdf 1105 KB) [file 10162_2025_999_MOESM1_ESM.pdf]

Supplementary Material

Figures

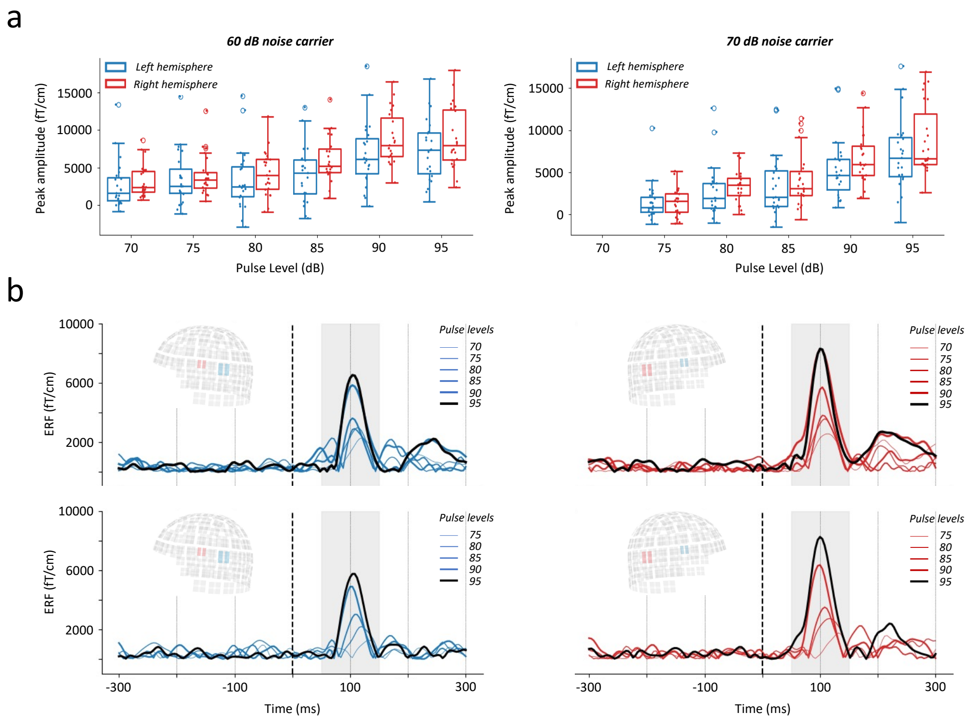

Supplementary Figure 1: A. Distribution of peak amplitude values in the sensors located at left (blue) and right (red) hemisphere for multiple pulse levels within 70 and 95 dB. Left and right panels display peak amplitude values with 60 and 70 dB broadband background noise carrier levels, respectively. (**b**) Grand averaged ERF responses are plotted per pulse level starting from 70 to 95dB SPL in steps of 5 dB in both 60 (upper panels) and 70 dB (bottom panels) broadband background carrier noise levels. Highest intensity responses (95 dB SPL) are colored in black. Box plots show individual values together with median ± 1.5 x IQR.

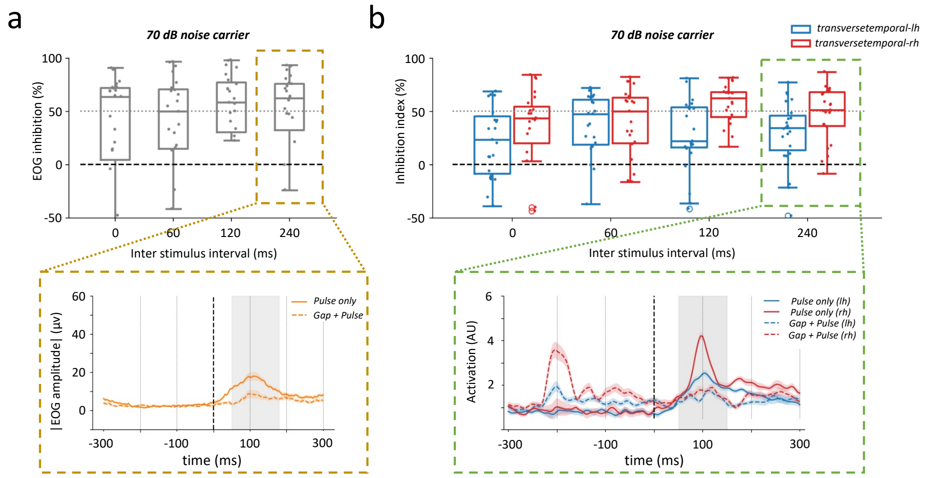

Supplementary Figure 2: A. Distribution of the EOG inhibition index values in the 70 dB broadband background noise carrier level, using the 90 dB pulse as the baseline condition. Each dot represents the ratio between the area under the curve when a gap coincided with the pulse and when there was no gap for various gap levels. Grand averaged EOG response for the pulse only and pulse followed by a 240 ms gap are plotted in the lower panel. (**b**) Boxplots illustrating distribution of inhibition index values computed in left and right transverse temporal gyrus for different levels of inter stimulus interval (ISI). Inhibition indices are computed as the ratio of area under the curve with and without gap presence at 70 dB broadband background noise carrier. Grand averaged activation in left (blue) and right (red) transverse temporal gyrus are plotted for the pulse only and pulse followed by a 240 ms gap in the lower panel. Box plots show individual values together with median ± 1.5 x IQR.

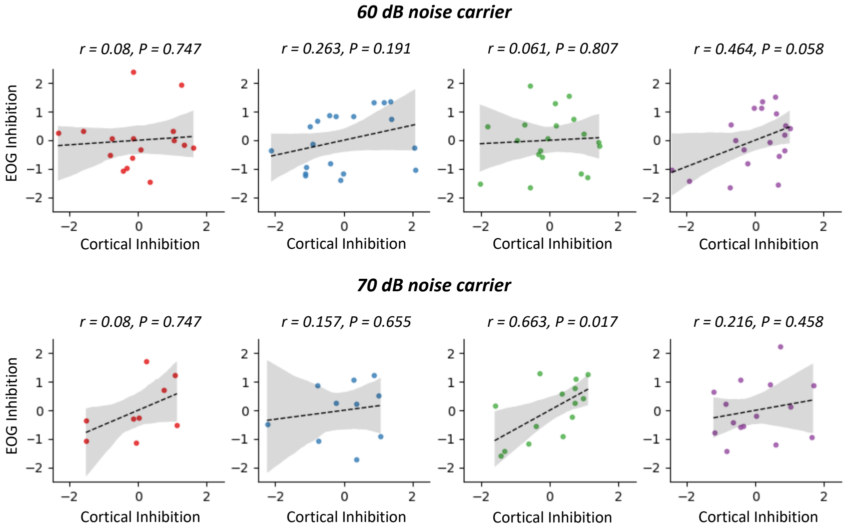

Supplementary Figure 3: Scatter plots, along with the regression line indicating the relationship between EOG inhibition and the inhibition index at the right transverse temporal gyrus, are depicted. Grey shaded areas represent 95% confidence intervals for the regression line and the plots represent gap durations of 0, 60, 120, and 240 ms from left to right, with 60 dB broadband background noise carrier levels. Only positive EOG responses are included in each plot (n = 10-20), while negative ones were excluded.

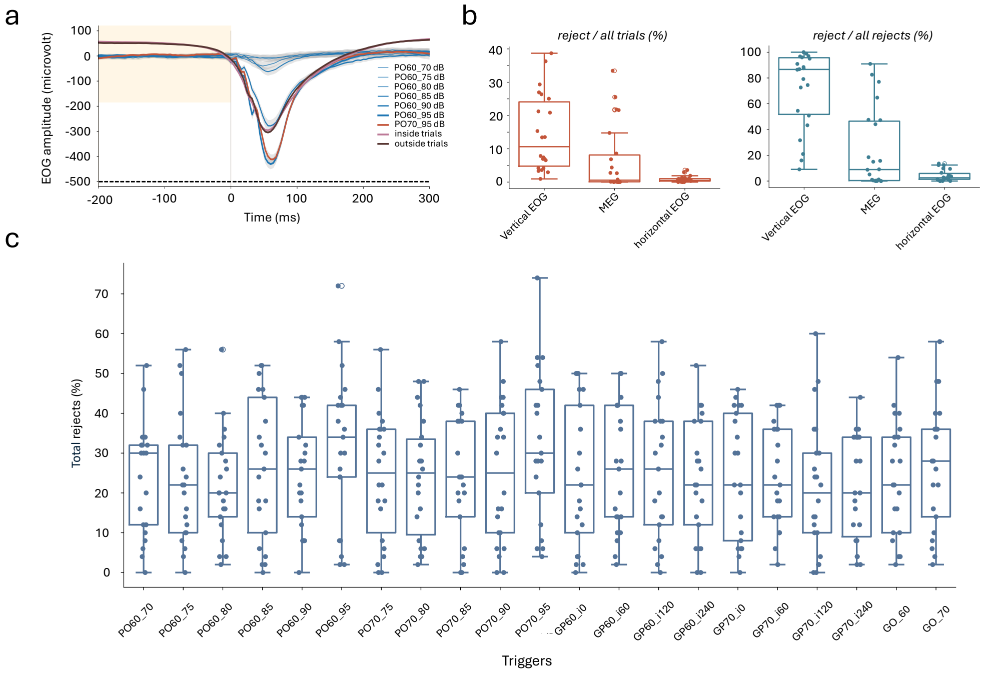

Supplementary Figure 4: a Averaged EOG signals recorded from a single participant are shown for both within-trial and between-trial periods, along with responses to auditory pulses delivered at 60 dB and the loudest pulse at 70 dB background noise. In this participant, vertical EOG amplitudes never exceeded $500\mu v$, indicating that spontaneous blinks in terms of amplitude are comparable to reflexive EOG responses across the full range of pulse intensities. **b** The left panel illustrates the proportion of trials excluded due to vertical and horizontal EOG activity, as well as MEG-related artifacts. The right panel shows the relative contribution of each rejection criterion to the total number of rejected trials (each dot represents one participant). **c** Percentage of total rejection rates for each trial type are shown, calculated as $1-(\frac{\text{number of retained trials}}{\text{total number of trials}})$. The total proportion of rejected trials (sum of the proportion of vertical, horizontal EOG rejects and the MEG rejects from panel (**b**) corresponds exactly to the sum of rejections across trial types in panel **c**, given that each trial type was repeated 50 times.

Tables

Supplementary Table 1: The results of the Tukey HSD (Honestly Significant Difference) multi-comparison test are presented for comparisons within different pulse levels at each broad band noise (BBN) carrier noise levels. Results refer to Figure 3. Significant p values < 0.05 are marked in bold.

Multiple Comparison of Means Over Pulse Levels							
PL (dB)		Adjusted p-value					
		60 dB BBN			70 dB BBN		
		EOG	LH	RH	EOG	LH	RH
75 dB	80 dB	0.9978	0.4009	0.7949	1.0	0.3109	0.1636
75 dB	85 dB	0.9802	0.1275	0.0592	0.9994	0.0625	0.0523
75 dB	90 dB	**0.0373**	**0.0001**	**3.76e-8**	0.3484	**0.0007**	**2.09e-7**
75 dB	95 dB	**0.0001**	**0.0001**	**2.93e-5**	**3.99e-5**	**4.08e-7**	**2.69e-11**
80 dB	85 dB	0.9992	0.9712	0.5082	0.9978	0.9404	0.9881
80 dB	90 dB	0.0830	**0.0491**	**5.99e-6**	0.2999	0.1941	**0.0022**
80 dB	95 dB	**0.0002**	**0.0437**	**0.0021**	**2.74e-5**	**0.0011**	**1.39e-6**
85 dB	90 dB	0.1452	0.2031	**0.0029**	0.4811	0.6164	**0.0111**
85 dB	95 dB	**0.0006**	0.1861	0.1780	**0.0001**	**0.0139**	**1.17e-5**
90 dB	95 dB	0.3490	1.0	0.5664	**0.0268**	0.3724	0.3508
